# Imaging in Gastroenterology

**DOI:** 10.1155/2014/963498

**Published:** 2014-05-11

**Authors:** Radu Badea, Iwona Sudoł-Szopińska, Sebastian Mueller, Horia Ştefănescu, Monica Lupsor Platon

**Affiliations:** ^1^Department of Clinical Imaging, “Octavian Fodor” Regional Institute of Gastroenterology and Hepatology and “Iuliu Hatieganu” University of Medicine & Pharmacy Cluj Napoca, Romania; ^2^Department of Radiology, Institute of Rheumatology, Warsaw and Department of Diagnostic Imaging, Warsaw Medical University, Poland; ^3^Department of Medicine, Salem Medical Center and Center for Alcohol Research, University of Heidelberg, Germany; ^4^Department of Hepatology, “Octavian Fodor” Regional Institute of Gastroenterology and Hepatology, Cluj Napoca, Romania

Imaging becomes more and more important for all clinical specialities, including gastroenterology. This issue is focusing on some very interesting and new applications of imaging procedures in liver, biliary tract, and digestive tube pathology. The content is representative for this “new era” of visual diagnosis that we live.

In the article entitled “*Preliminary study on hepatocyte-targeted phosphorus-31 MRS using ATP-loaded galactosylated chitosan oligosaccharide nanoparticles*” R.-S. Yu et al. are describing their preliminary work on MR spectroscopy for evaluating the hepatocyte uptake of ATP-loaded Gal-CSO (Gal-CSO/ATP) nanoparticles. They show significant targeting efficiency in hepatic cells in vitro and enhancement efficiency of ATP peaks in HepG-2 cells. Furthermore, they demonstrate that phosphorus-31 MR spectroscopy could be applied in the research of hepatic molecular imaging.

V. Granata et al. in the article “*Surveillance of HCC patients after liver RFA: role of MRI with hepatospecific contrast versus three-phase CT scan*—*experience of high volume oncologic institute*” compare the diagnostic accuracy of hepatospecific contrast-enhanced MRI versus triple-phase CT scan after radiofrequency ablation (RFA) in hepatocellular carcinoma (HCC) patients. This is an excellent paper with a very good design. The authors examine a cohort of thirty-four consecutive HCC patients (42 hepatic nodules), treated with percutaneous RFA, who underwent MR and CT scan. All patients were enrolled in a research protocol that included CT with iodized contrast medium injection and MR with hepatospecific contrast medium injection and restaged within four weeks and at 3 months after ablation. Following this work, hepatospecific contrast-enhanced MRI seems to be more effective than multiphase CT in assessment of HCC treated with RFA.

T. Gorycki et al. in the article entitled “*Bile duct strictures caused by solid masses: MR in differential diagnosis and as a prognostic tool to plan the endoscopic treatment*” assess the meaning of qualitative parameters counted from magnetic resonance (MR) plane images as well as from cholangiopancreatography (MRCP) including cases of bile duct obstructions caused by solid masses in differential diagnosis between benign and malignant conditions and as the prognostic factors for endoscopic treatment. They conclude that the probability of malignancy of solid lesions obstructing biliary duct is increasing with higher SIR in T2W images and with longer strictures. Passing the stricture during ERCP treatment was easier and more probable in cases of shorter strictures caused by lesions with higher SIR in STIR T2W images.

B. Małkowski et al. make a study entitled “^*18*^ *F-FLT PET/CT in patients with gastric carcinoma*” focused on gastric carcinoma. Gastric cancer is a neoplasm presenting different types, frequently containing mucus. Cellularity of this tumor type can be limited and ^18^F-glucose uptake is not high enough to be seen in PET. The aim of the study was to evaluate the usefulness of ^18^F-FLT PET/CT in the detection and differentiation of gastric cancers. The work suggests that there is a good accumulation of fluorothymidine in such a tumor which enabled the visualization and evaluation. Simultaneously to neoplastic cells fluorothymidine was accumulated in normal mucosa, so the authors analyzed optimal cut-off value for SUVmax in normal and neoplastic tissue. Ultrasonography (especially EUS) plays the important role in gastric cancer staging but it should be used together with other imaging modalities (e.g., PET) indicating possible invasion of any anatomical and pathological structures.

The enterovesical fistulas (the paper entitled “*Enterovesical fistulae: aetiology, imaging, and management*”) are discussed in the report made by T. Golabek et al. The study provides a state-of-the-art overview of the clinical and radiological diagnosis of enterovesical fistulae. The treatment of fistulae is also briefly discussed. The diagnosis of EVF can be challenging and is often delayed for several months after the symptoms begin. Computed tomography and magnetic resonance imaging remain ideal modality options in establishing the site, course, and complexity of fistulae and in identifying an aetiological factor. Management of fistulae is mainly dependent on the underlying pathology, site of the bowel lesion, and patient's preoperative performance status and may involve conservative or invasive treatment. However, surgical one-stage strategy is a preferred option in most of the cases.

Dr. W. Memon et al. in the paper entitled “*MDCT of small bowel obstruction: how reliable are oblique reformatted images in localizing point of transition?*” evaluate prospectively the additional value of oblique reformations from isotropic voxels obtained using a 64-slice MDCT (multidetector row CT) to localize POT (point of transition), having surgery as a reference standard. Early diagnosis of SBO (small bowel obstruction) is essential to prevent bowel ischemia. Although plain X-ray abdominal evaluation still remains the investigation of first choice in cases of suspected SBO due to its low cost and wide availability, it cannot reliably diagnose the exact level of obstruction and thus can only serve as a basis for triage for further imaging workup. With the ongoing developments in imaging techniques overtime, computed tomography (CT) has emerged as an excellent modality in the diagnosis of SBO. CT scan is not only reliably diagnose SBO but also can be of great help in determining the cause, severity, and the precise point of obstruction. Localizing the point of transition (POT) is empirical as it increases confidence in diagnosis, guides patient care, and thus helps in further management. The authors found that although the oblique MPR increases the image load and is time consuming it surely represents a development and new perspective of utilizing the available information whereby it can significantly prove as a powerful adjunct in diagnosis and management of SBO.

Dr. T. S. Kirtane and M. S. Wagh in the work entitled “*Endoscopic optical coherence tomography (OCT): advances in gastrointestinal imaging*” present the current status of OCT and its practical applications in imaging normal and abnormal mucosa in the esophagus, stomach, small and large intestines, and biliary and pancreatic ducts. They also highlight technical aspects and principles of imaging, assess published data, and suggest future directions for OCT-guided evaluation and therapy.

Finally, the group of Dr. A. Gebesce et al. (in the work entitled “*Importance of the ultrasonography in diagnosis of ileal duplication cyst*”) are presenting an interesting case of intestinal duplication cyst and are discussing the role of ultrasonography for the diagnosis of this rare condition, both ante- and postpartum.

In conclusion, the present issue represents a holistic approach of the visual diagnosis in modern gastroenterology.

*Radu Badea* *Radu Badea* *Iwona Sudoł-Szopińsk* *Iwona Sudoł-Szopińsk* *Sebastian Mueller* *Sebastian Mueller* *Horia Ştefănescu* *Horia Ştefănescu* *Monica Lupsor Platon* *Monica Lupsor Platon*

